# Construction and validation of a prognostic marker and risk model for HCC ultrasound therapy combined with WGCNA identification

**DOI:** 10.3389/fgene.2022.1017551

**Published:** 2022-10-03

**Authors:** Yunlong Bi, Yu Jing, Lingling Guo

**Affiliations:** ^1^ Department of Orthopedics, First Affiliated Hospital of Jinzhou Medical University, Jinzhou, China; ^2^ Department of Oncology, First Affiliated Hospital of Jinzhou Medical University, Jinzhou, China; ^3^ Department of Ultrasound, First Affiliated Hospital of Jinzhou Medical University, Jinzhou, China

**Keywords:** hepatocellular carcinoma (HCC), ultrasound, WGCNA, prognosis, risk model

## Abstract

**Background:** Hepatocellular carcinoma (HCC) is a malignant tumor with a highly aggressive and metastatic nature. Ultrasound remains a routine monitoring tool for screening, treatment and post-treatment recheck of HCC. Therefore, it is of great significance to explore the role of ultrasound therapy and related genes in prognosis prediction and clinical diagnosis and treatment of HCC.

**Methods:** Gene co-expression networks were developed utilizing the R package WGCNA as per the expression profiles and clinical features of TCGA HCC samples, key modules were identified by the correlation coefficients between clinical features and modules, and hub genes of modules were determined as per the GS and MM values. Ultrasound treatment differential expression genes were identified using R package limma, and univariate Cox analysis was conducted on the intersection genes of ultrasound differential expression genes and hub genes of key HCC modules to screen the signatures linked with HCC prognosis and construct a risk model. The median risk score was used as the threshold point to classify tumor samples into high- and low-risk groups, and the R package IOBR was used to assess the proportion of immune cells in high- and low-risk groups, R package maftools to assess the genomic mutation differences in high- and low-risk groups, R package GSVA’s ssgsea algorithm to assess the HALLMARK pathway enrichment analysis, and R package pRRophetic to analyze drug sensitivity in patients with HCC.

**Results:** WGCNA analysis based on the expression profiles and clinical data of the TCGA LIHC cohort identified three key modules with two major clinical features associated with HCC. The intersection of ultrasound-related differential genes and module hub genes was selected for univariate Cox analysis to identify prognostic factors significantly associated with HCC, and a risk score model consisting of six signatures was finally developed to analyze the prognosis of individuals with HCC. The risk model showed strength in the training set, overall set, and external validation set. The percentage of immune cell infiltration, genomic mutations, pathway enrichment scores, and chemotherapy drug resistance were significantly different between high- and low-risk groups according to the risk scores. Expression of model genes correlated with tumor immune microenvironment and clinical tumor characteristics while generally differentially expressed in pan-cancer tumor and healthy samples. In the immunotherapy dataset, patients in the high-risk group had a worse prognosis with immunotherapy, indicating that subjects in the low-risk group are more responsive to immunotherapy.

**Conclusion:** The 6-gene signature constructed by ultrasound treatment of HCC combined with WGCNA analysis can be used for prognosis prediction of HCC patients and may become a marker for immune response.

## Introduction

Liver cancer is among the leading causes of fatalities resulting from malignancies around the globe, and hepatocellular carcinoma (HCC) is the most prevalent kind of primary HCC, covering 90% of all primary liver cancer (Jafri and Kamran, 2019). In China, the incidence and mortality of HCC rank fourth and third, respectively, among malignant tumors, with a very high degree of malignancy (Chen et al., 2016). Individuals with HCC have a poor prognosis, with a 5-year survival rate of fewer than 18% (Forner et al., 2018; Siegel et al., 2020). Currently, systemic chemotherapy is an important treatment for patients with advanced HCC who have not undergone surgical resection, local radiofrequency ablation, or selective arterial chemoembolization. However, chemotherapeutic drugs are often associated with greater drug resistance and serious systemic toxic adverse effects. Therefore, developing a safe and effective drug delivery system is crucial.

Ultrasound is a routine monitoring tool for screening and post-treatment re-examination of HCC (Chen et al., 2019). With the advancement of ultrasound molecular imaging technology and its application in the clinic, people are now able to use this technology to diagnose and treat patients more accurately, which is expected to break through the treatment failure caused by chemotherapy resistance. Studies have shown that ultrasound microbubbles can not only enhance imaging but also serve as a novel drug delivery vehicle to achieve local targeted drug delivery by breaking microbubbles through local ultrasound irradiation, resulting in increased local drug concentrations and reducing systemic toxic adverse effects of drugs (Jang et al., 2020; Omata et al., 2020; Tian et al., 2021). The cavitation and acoustic pore effects generated during the breakdown of microbubbles by ultrasound irradiation can directly affect tumor tissues and destroy tumor blood vessels, leading to apoptosis of tumor cells and inhibiting tumor growth (Fan et al., 2016; Jing et al., 2016; Chowdhury et al., 2020). Therefore, exploring the rationale and biological significance of ultrasound technology in HCC to affect prognostic survival can further exploit the role of this technology in tumor treatment.

In this study, we obtained a collection of co-expressed ultrasound differential genes that correlate clinical features and survival in HCC by collecting expression data from HCC samples in TCGA and GEO datasets, facilitating WGCNA analysis and differential expression analysis. The association between this gene collection’s expression perturbation and the prognosis for HCC prognosis was investigated at multiple levels. Subsequently, a risk score model for evaluating the prognosis of HCC was developed, and the stable efficacy of the model for prognostic assessment was confirmed.

## Materials and methods

### Dataset source and preprocessing

Expression profile data (FPKM values) and clinical data of Liver Hepatocellular Carcinoma (LIHC) from The Cancer Genome Atlas database (TCGA) were downloaded using the R package TCGAbiolinks. The FPKM values underwent log2 transformation, while a uniform unit of survival time: “days”, was used to process the survival information.

We downloaded the expression profile and ultrasound grouping information of GSE178573, expression data, as well as clinical information of GSE14520, GSE76427 and LIRI-JP from GEO (https://www.ncbi.nlm.nih.gov/geo/) database and subsequently, proceeded with the following steps: 1) Removed the samples with no data on clinical follow-up; 2) removed the samples with unknown survival time, less than 0 days, or no survival status, and unified the survival time unit as days; 3) converted the probes to Gene Symbol; 4) removed one probe corresponding to multiple genes; 5) took the median value for expression cases with multiple Gene Symbols. Expression profiles and survival and response information for the IMvigor210 immunotherapy cohort (bladder cancer) were downloaded using the R package IMvigor210CoreBiologies.

The immunotherapy dataset for clear cell carcinoma was downloaded from published literature (Braun et al., 2020).

### WGCNA analysis

Weighted gene co-expression network analysis (WGCNA) separates the gene co-expression network of complex biological processes into highly linked signature modules, which represent various sets of highly synergistic gene sets. This technique enables the association of modules with particular clinical characteristics for finding genes that have important roles, assisting in the identification of potential mechanisms underlying certain specific biological processes as well as exploring candidate biomarkers. Gene co-expression networks were developed with the help of the R package WGCNA as per the expression profiles and clinical features of TCGA HCC samples, and key modules were identified by the correlation coefficients between clinical features and modules. The hub genes of the modules were then identified based on GS and MM values, after which the co-expression network maps of the hub genes were constructed using cytoscape software.

### Differential expression analysis

Using the R package limma, differential expression analysis was carried out. The Benjamini–Hochberg (FDR) corrected *p*-value adj. *p* value<0.05 and |log2FC|> 0.585 were used as thresholds to identify differentially expressed genes.

### Prognostic risk modeling and survival difference analysis

The intersection of ultrasound differentially expressed genes and hub genes of key modules of HCC was subjected to univariate cox analysis to screen (*p* < 0.05) signatures associated with HCC prognosis. Meanwhile, LIHC samples were split into groups with high and low expressions of signature expressions using the median expression of each signature as the cutoff point. Survival curves for prognostic analysis were then generated using the Kaplan-Meier method, and the significance of differences was assessed utilizing the log-rank test. In order to build a prognostic model, the main prognosis-related genes were then further evaluated using the LASSO regression method of the R package glmnet. The tumor samples were categorized into high and low-risk groups by means of the median risk score as the cutoff point. Kaplan-Meier survival curves were then created for prognostic analysis, and the significance of the differences was observed using the log-rank test. The receiver operating characteristic (ROC) curves were then plotted using the R package timeROC for evaluating the prediction of scoring by the perturbation scoring model; the R package ggplot2 was employed for creating the scatter plot of survival time *versus* survival status, and the scatter plot of sample scores; the R package pheatmap was utilized for plotting the expression thermographic of model genes, where the risk value of the model is the summation of individual candidate gene expression value multiplied by the weight, with the following equation.

$$RiskScore=\overset{n}{\underset{i=0}{\sum }}coef\left(i\right)*\mathrm{Exp}\left(i\right)$$

### Immune infiltrating cell proportion estimation and immune scoring

Four algorithms from the R package IOBR, TIMER, ESTIMATE, xCell, and CIBERSORT, were used to determine the proportion of immune infiltrating cells based on the expression patterns of the TCGA LIHC dataset. The CIBERSORT algorithm (Newman et al., 2015) is a method to describe the cell composition of complex tissues according to their gene expression patterns. The identification of 22 immune cell types, including myeloid subpopulations, natural killer (NK) cells, plasma cells, naïve and memory B cells, and seven different types of T cells, was done using the leukocyte signature gene matrix LM22, which consists of 547 genes. CIBERSORT combined with the LM22 signature matrix was used for estimation of the proportion of the 22 kinds of cell phenotypes in the samples, with the sum of all immune cell types’ proportions in individual samples being equal to 1.

The ESTIMATE algorithm was employed to determine the immune score, tumor purity, matrix score and ESTIMATE score for tumors. xCell conducts cell type enrichment analysis using data on the 64 immune and stromal cell types’ gene expression. In order to minimize the correlation between closely linked cell types, xCell employs machine learning based on gene signatures from thousands of different cell types. By validating extensive computer simulations of signature and cellular immunophenotyping, xCell is able to reliably map the cellular heterogeneity of tissue expression profileslandscape. TIMER uses an inverse convolution approach for estimating the proportion of six immune cell types in 32 cancers (neutrophils, CD4^+^ T cells, CD8^+^ T cells, B cells, dendritic cells, and macrophages). Online gene searches were also used to investigate the relationship between the expression of model genes (TPM) and the proportion of immune infiltrating cells, as well as the differences between the expression of model genes in pan-cancerous tumors and normal tissue.

### Genomic mutation analysis

Waterfall plots were drawn using the R package maftools combined with clinical grouping information to demonstrate the distribution of mutations in genes with high somatic mutation frequencies in HCC samples, and waterfall plots were also drawn with model grouping information to classify the samples.

### HALLMARK pathway enrichment analysis

The ssgsea algorithm of R package GSVA was utilized for calculating 50 HALLMARK pathway enrichment scores for each sample on the basis of gene expression of HCC samples. The correlation between the riskscore and the enrichment score was measured using the cor function and visualized with the R package corrplot. Enrichment score differences between model subgroups were then calculated using statistical tests, and enrichment score thermographics were plotted by the R package pheatmap along with the clinical characteristics of the samples. Drug sensitivity analysis was done utilizing the R package pRRophetic, combined with expression data of model genes, for predicting the sensitivity (IC50 values) of 138 drugs in the GDSC database and the sensitivity of HCC patients to drug treatment was assessed by IC50 values. The differences in IC50 values between the risk groups were compared by the Wilcoxon test, and drugs with major variations in the two groups were screened.

### Statistical tests

For significance labeling, the Wilcoxon test was employed for comparison of variations between two groups of samples, while Kruskal–Wallis was employed for comparison of the variations between multiple groups of samples. Where ns denotes *p* > 0.05, * denotes *p* <= 0.05, ** denotes *p* <= 0.01, *** denotes *p* <= 0.001, and **** denotes *p* <= 0.0001. Among which *p* < 0.05 shows a significant difference.

## Results

### WGCNA identification of key traits and modules in HCC

**FIGURE 1:**
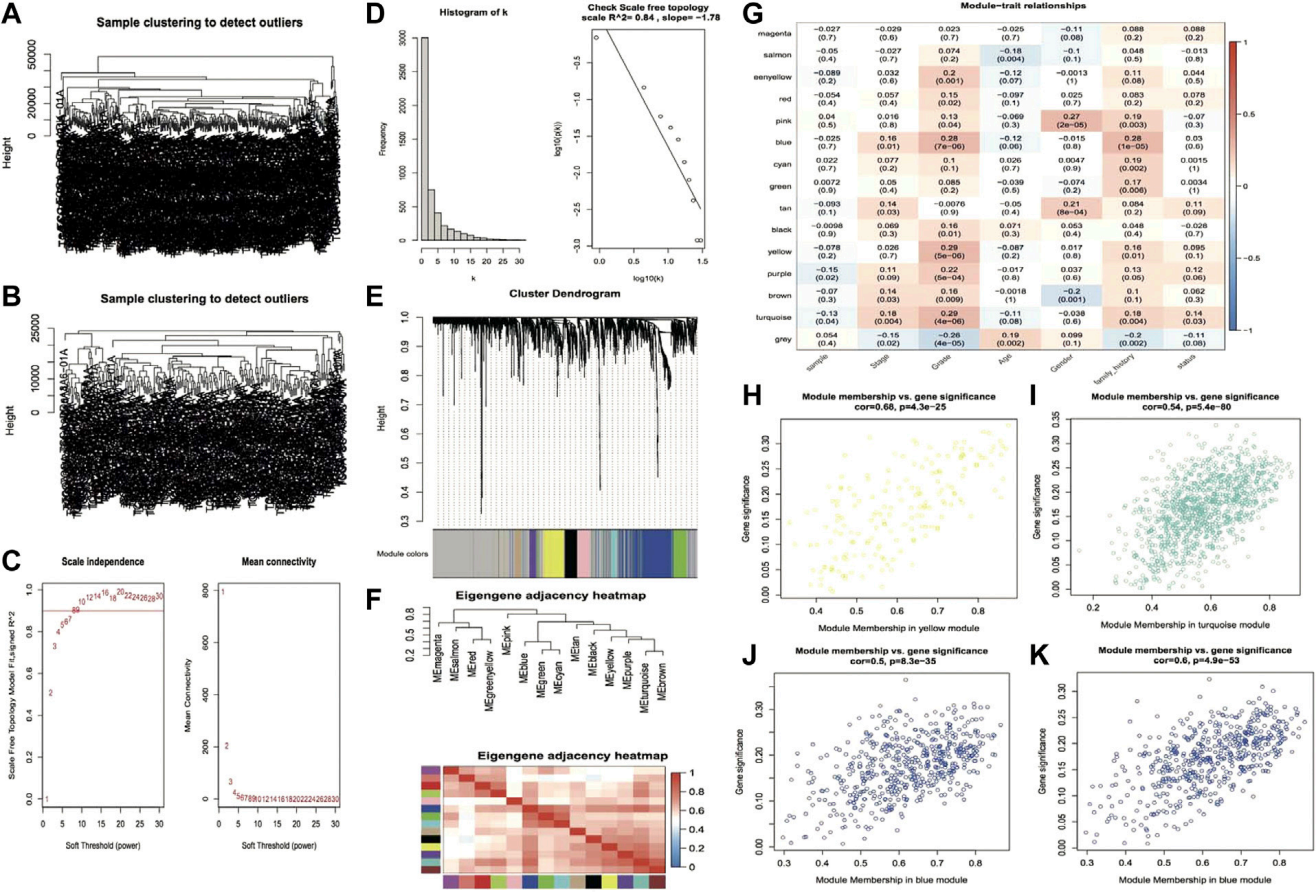
The plot of the results of weighted gene co-expression network analysis. **(A,B)** Clustering tree of samples before and after outlier subjects removal; **(C)** Soft threshold distribution scatter plot, Soft Threshold (power) indicates the weight, vertical coordinate indicates the correlation of connectivity k with *p*(k) and average connectivity; **(D)** Soft Threshold test plot; **(E)** Clustering tree of genes within modules, the top half of the plot is the clustering tree of genes, the bottom half is the modules clustered according to similarity; **(F)** Eigenvector gene clustering tree and module correlation thermographic; **(G)** Module trait correlation thermographic; **(H–K)** Scatter plots of GS and MM value distribution within modules, rows represent modules, columns are traits, and values are correlation coefficients.

The genes with the top 5,000 variants in the expression profile of the TCGA LIHC cohort were selected for WGCNA analysis. Initially, 363 HCC samples were clustered, and the results are shown in Figure 1A. Afterward, the cutHeight was set to 28,000 to eliminate the outlier samples, and finally, 247 samples were obtained for subsequent analysis, and the clustering tree after eliminating the outlier samples is shown in Figure 1B. When the correlation coefficient is >0.8, the optimal soft threshold is set as 7 (Figure 1C). Furthermore, the memory network was checked to see if it approximates scale free with the chosen β value. From Figure 1D, we can see that k is negatively linked with *p* (*k*) (correlation coefficient = 0.84), suggesting that the chosen β value is capable of establishing a gene-free scale network. Then the minimum gene number within the module was set to 30, and the maximum module distance was 0.25. Subsequently, the Pearson correlation method was used to calculate the co-expression correlation and the module trait correlation and construct the co-expression network. From the module clustering tree, we can see that yellow and blue are important modules (Figure 1E). Then the eigenvector gene clustering tree and thermographic were plotted, and the results showed modules with correlation coefficients >0.8 (dissimilarity coefficient <0.2) (Figure 1F), which were merged in the subsequent analysis. The module-trait correlation thermographic is shown in Figure 1G, which shows the key traits (grade and family history) and the key modules (yellow, turquoise, and blue). Scatter plots are then drawn to show the linear relationship between GS and MM within modules, and the results are shown in Figures 1H–K: the correlation coefficients are 0.68, 0.54, 0.5, and 0.6.

### Co-expression network and enrichment analysis of hub genes in key modules

**FIGURE 2:**
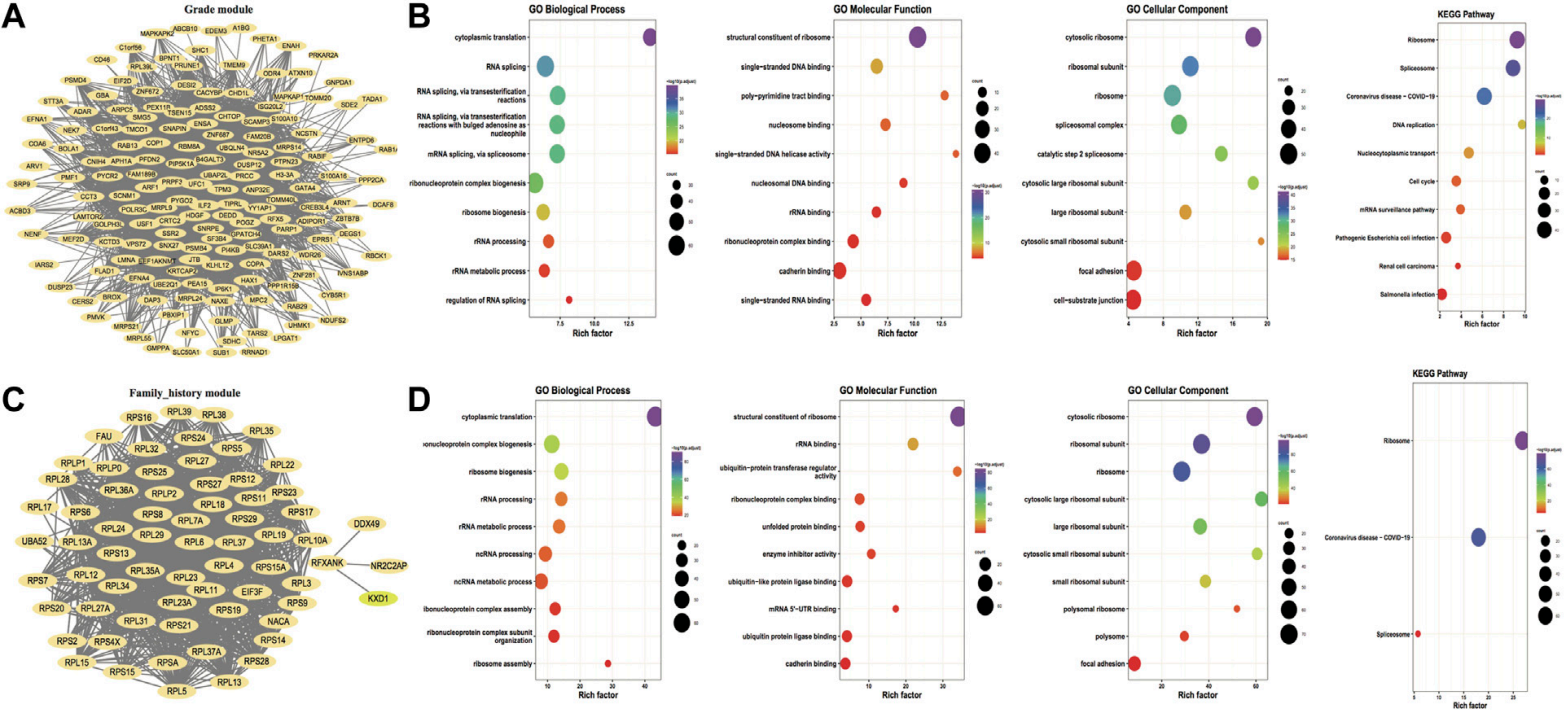
Hub gene co-expression network and hub gene enrichment analysis. **(A,C)** Co-expression network of hub genes for key traits grade and family_history, respectively, and the nodes of both FromNode and ToNode are selected for hub genes; **(B,D)** Functional enrichment analysis and KEGG pathway enrichment for key traits Grade and family_history, respectively. The dot’s size demonstrates the number of enriched hub genes and the color demonstrates the significance of enrichment.

In accordance with the distribution of GS and MM values of genes in the modules, a threshold value of GS > 0.2 and MM > 0.6 was set to identify hub genes for key modules of each key trait Moreover, the three key modules of grade trait and the blue module of family history were screened to obtain 467 hub genes and 200 hub genes, respectively. Then the hub genes were screened based on the edge and node files obtained from the exportNetworkToCytoscape function in WGCNA and imported into Cytoscape to construct the module hub gene co-expression network maps for the key traits; the outcomes are illustrated in Figure 2A and Figure 2C. Then the GO function enrichment analysis and KEGG enrichment analysis were performed for the two hub gene sets, respectively, and the TOP 10 entries of the enrichment outcomes were chosen to draw bubble plots that are illustrated in Figure 2B and Figure 2D.

### Ultrasound-associated prognostic signature construction and validation

#### Screening of ultrasound-associated hub genes for HCC

**FIGURE 3:**
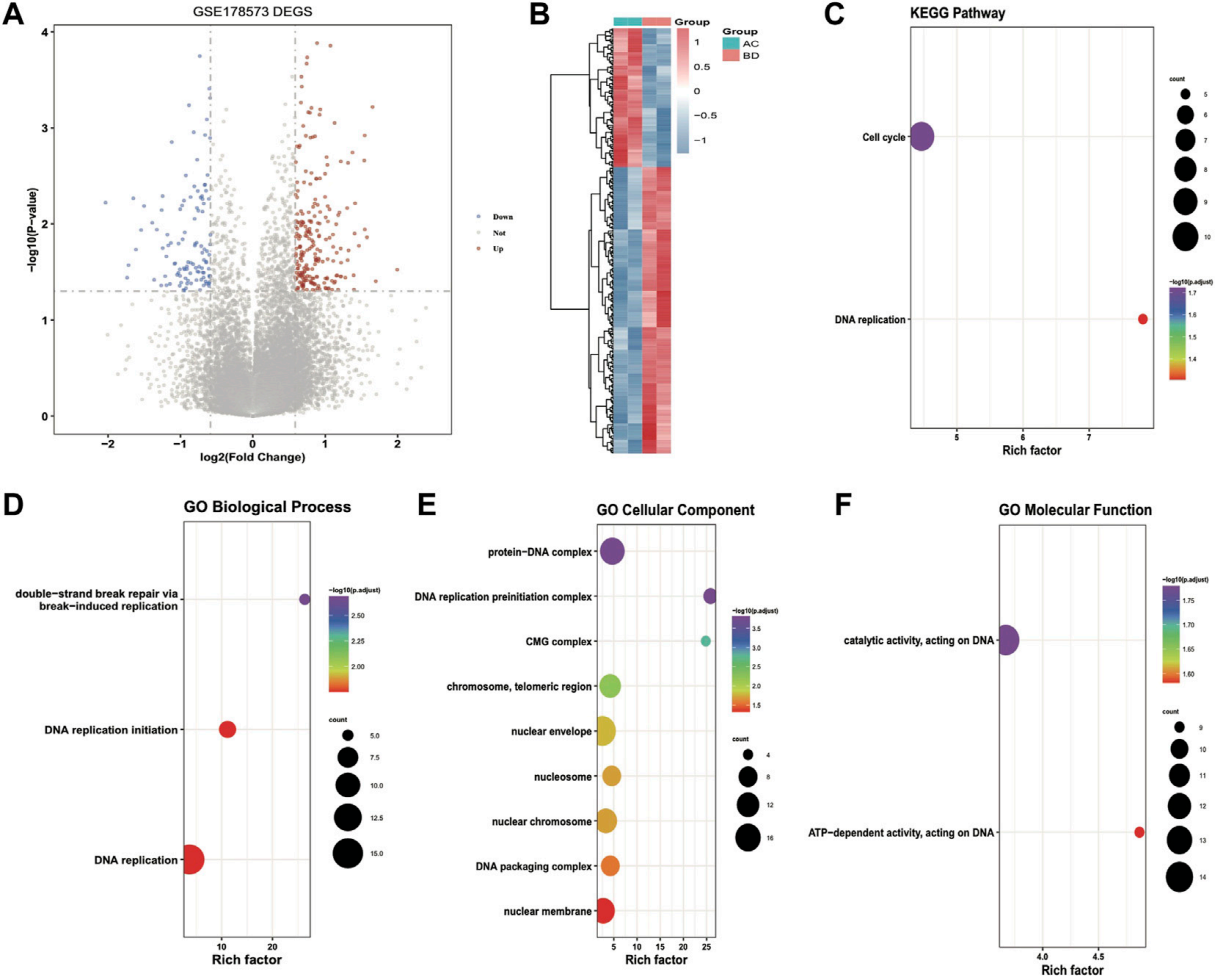
Results of differential expression analysis and functional enrichment analysis of GSE178573 dataset. **(A)** volcanic plot of differentially expressed genes in ultrasound and non-ultrasound groups; **(B)** thermographic of differentially expressed genes, red highlights high expression, blue highlights low expression; **(C–F)** Enrichment of differentially expressed genes for KEGG, BP (biological process), CC (cellular component), MF (molecular function) Pathway bubble plots, where the dot’s size demonstrates the number of enriched differentially expressed genes, and the color demonstrates the significance of the enrichment results.

Differential expression analysis was performed on ultrasound and non-ultrasound samples from the GSE178573 dataset to screen ultrasound-related differentially expressed genes. Subsequently, 340 significantly differentially expressed genes were obtained, including 229 up-regulated genes and 111 down-regulated genes, and volcanic plots and thermographics were drawn to demonstrate the expression distribution of differentially expressed genes among subtypes; the outcomes are illustrated in Figure 3A, B. Subsequently, KEGG enrichment analysis and GO functional enrichment analysis were performed on the identified differentially expressed genes, and the outcomes are demonstrated in Figure 3C–F: the TOP 10 entries with significant enrichment outcomes were chosen to draw bubble plots, and the size of the dots demonstrate the number of enriched differentially expressed genes, and the color highlights the significance of enrichment. Then the hub genes of key modules of HCC obtained from WGCNA analysis were intersected with the ultrasound differentially expressed genes, and a total of 14 intersected genes were obtained, called the Module DEGs.

#### Module DEGs prognosis signature construction

**FIGURE 4:**
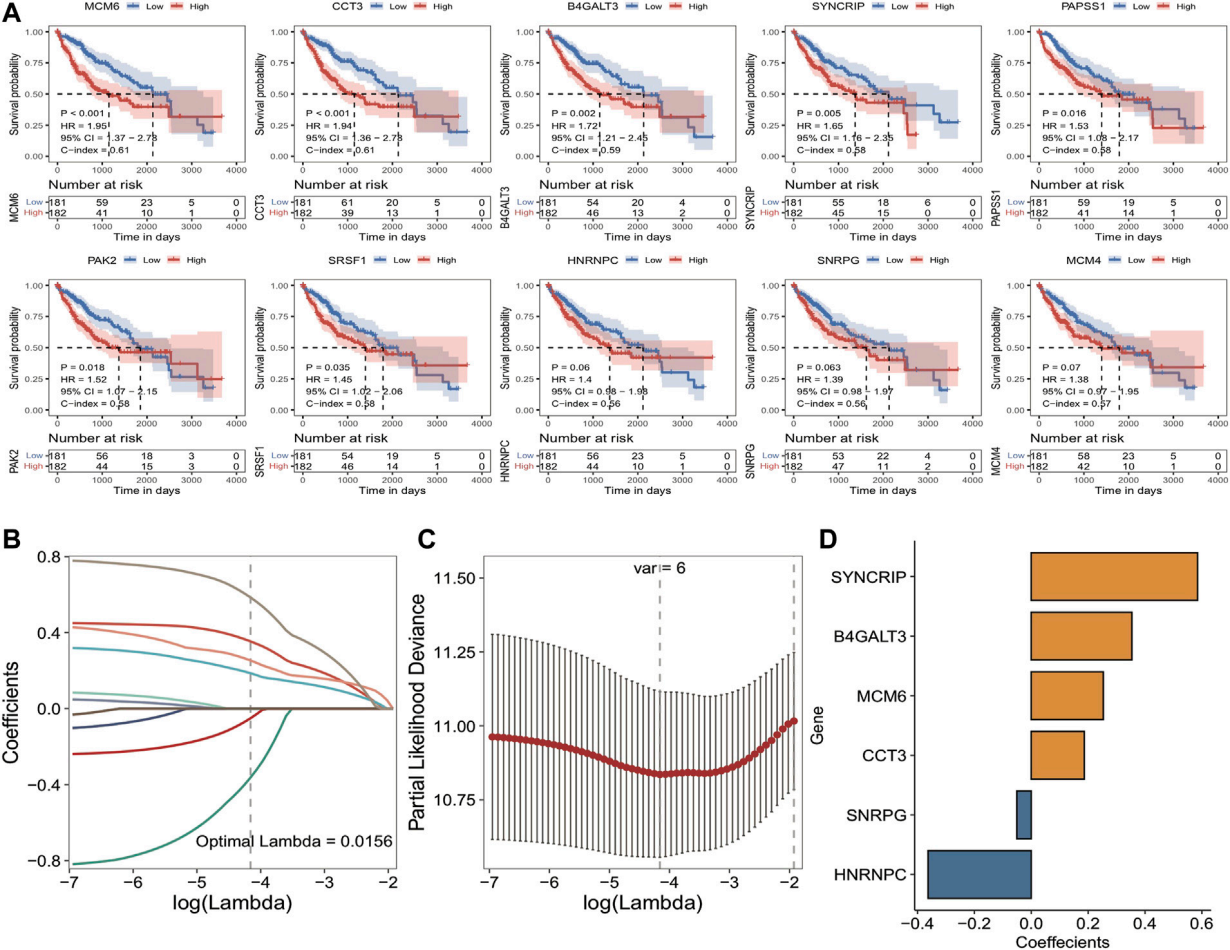
Results of TCGA training set cox analysis and Lasso regression analysis. **(A)** KM curves of prognostic signatures obtained from COX analysis; **(B)** Trajectories of the independent variables of LASSO regression, the horizontal coordinates indicate the logarithm of the independent variable Lambda, and the vertical coordinates indicate the coefficients of the independent variables; **(C)** LASSO regression under each Lambda confidence interval; **(D)** LASSO regression coefficients of key prognostic genes.

Subsequently, 2/3 of the overall TCGA_LIHC set (*n* = 363) was selected as the training set (*n* = 242) by random sampling, and 14 Module DEGs were screened in training set by means of univariate Cox analysis. *p* < 0.05 was set as the threshold to finally obtain ten genes associated with prognosis, and then the median expression of each gene was taken as the cutoff value for high and low grouping and to plot Kaplan-Meier survival curves, as illustrated in Figure 4A: major variations were observed in KM curves for 7 of these genes. Based on these ten prognosis-related signatures, set seed = 212,110, and using LASSO linear regression, redundant genes were removed, and a risk model was constructed; the results are shown in Figures 4B–D: 6 prognosis-related signatures were finally screened. The outcomes of Cox and Lasso analyses.

#### Internal validation set to check the strength of the risk model

**FIGURE 5:**
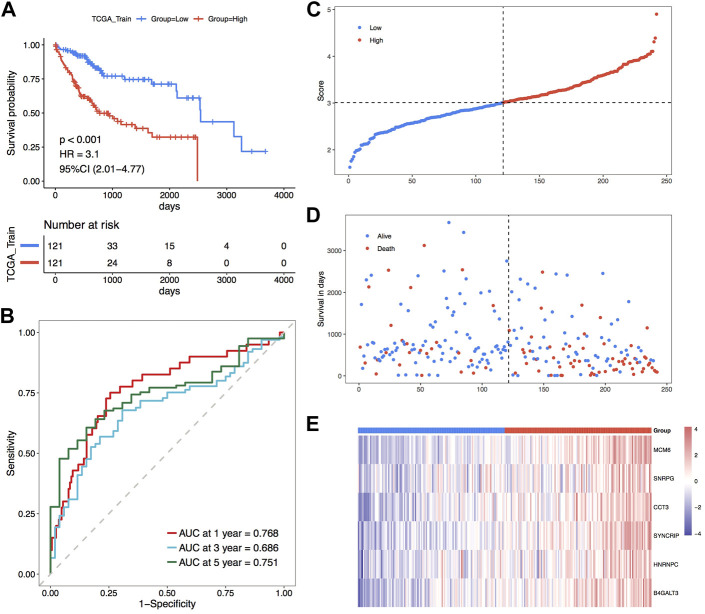
TCGA training set for the validation of the model’s prognostic efficacy. **(A)** KM curve of TCGA training set; **(B)** ROC curve; **(C–E)** Risk triple-plot, including scatter plot of risk score, scatter plot of survival time and thermographic of model gene expression in risk score grouping, red highlights high-risk group and blue highlights the low-risk group.

To further determine the impact of the model scores constructed from the six signatures on the overall survival of the training set. Initially, the median of RiskScore was used as the threshold value, the samples were sorted into high and low-risk groups, and KM curves were plotted, and the results showed that there was a major variation in prognosis between the high- and low-risk groups with a worse prognosis in the samples of high-risk group (Figure 5A). According to the constructed risk model, the ROC curves of prognostic signature were plotted, and the respective AUC values at 1, 3, and 5 years were 0.768, 0.686 and 0.751, indicating the good predictive accuracy of the model scores (Figure 5B). Moreover, scatter plots of survival time and status (Figure 5C) and scatter plots of sample risk scores (Figure 5D) were plotted, and the relationship between survival and RiskScore could be observed by combining these two scatter plots. Subsequently, the expression thermographic of model genes shows that model genes are highly expressed in the high-risk group of the training set (Figure 5E).

**FIGURE 6:**
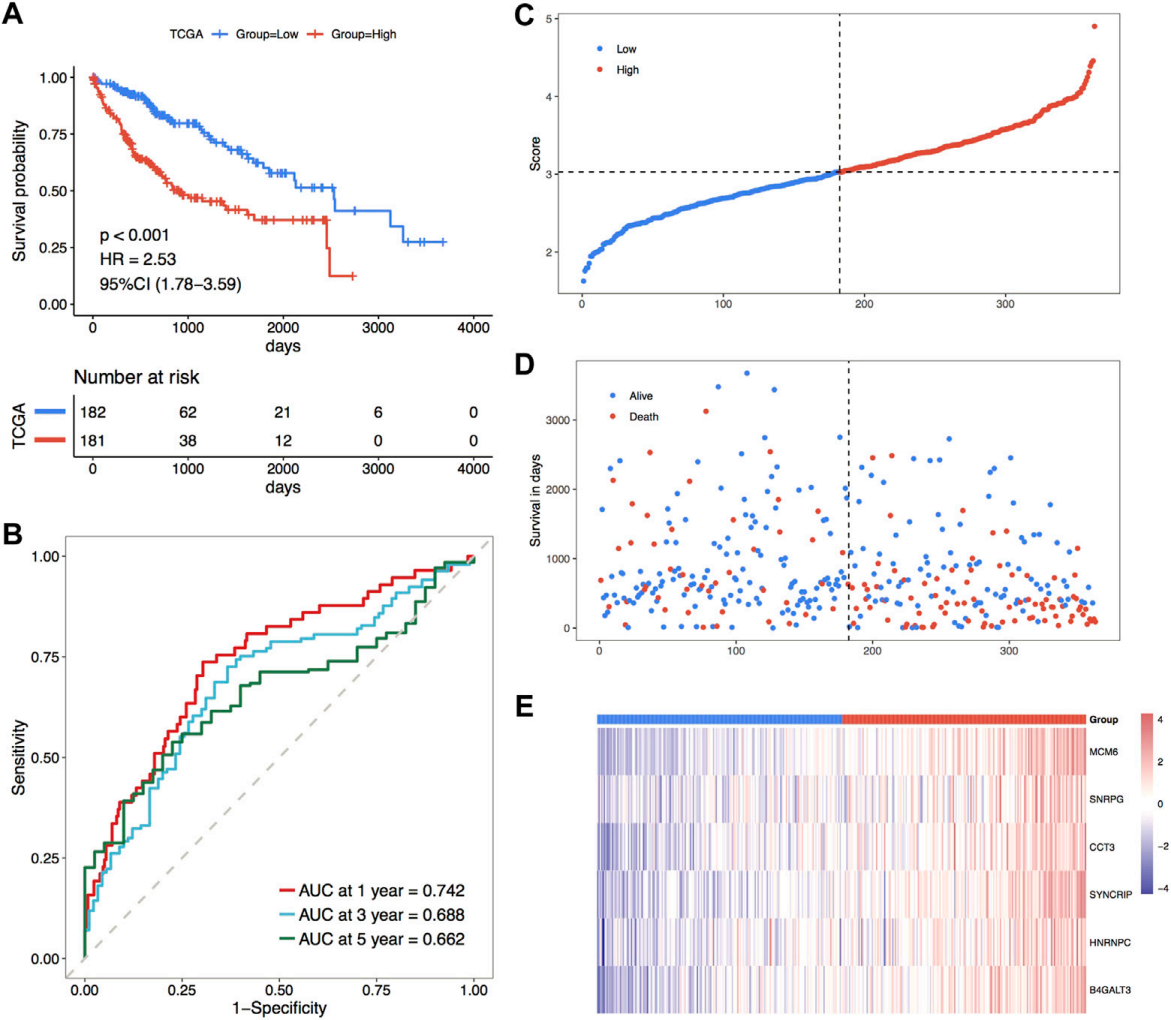
Prognostic efficacy of TCGA holistic set validation model. **(A)** KM curves of TCGA holistic set; **(B)** ROC curves of TCGA holistic set at 1, 3 and 5 years; **(C–E)** risk triple-plot plots with risk score scatter plot, survival time scatter plot and heat plot of model gene expression in risk score grouping, red highlights the high-risk group and blue highlights the low-risk group, respectively.

Subsequently, the overall set of TCGA_LIHC was used to test the predictive ability of RiskScore for overall survival. Based on the same method as the TCGA training set, the overall set samples were sorted into high and low-risk groups, with a worse prognosis observed in the high-risk group, and the prognosis of both groups varied substantially (Figure 6A). In the overall dataset of TCGA_LIHC, its respective AUCs at 1, 3, and 5 years were 0.742, 0.688 and 0.662 (Figure 6B). The scatter plots of the sample risk scores and the scatter plots of survival time and status for the two datasets are shown in Figures 6C, D, which highlight the risk score distribution among the samples. The expression thermographic of model genes in the corresponding dataset is shown in Figure 6E; therefore, it is clear that the distribution of gene expression in the samples of the dataset is consistent with the trend of expression in the validation set. The validation results of the overall TCGA set indicate that the model score has good and stable efficacy for survival prediction.

#### External validation sets to verify model prognostic efficacy

**FIGURE 7:**
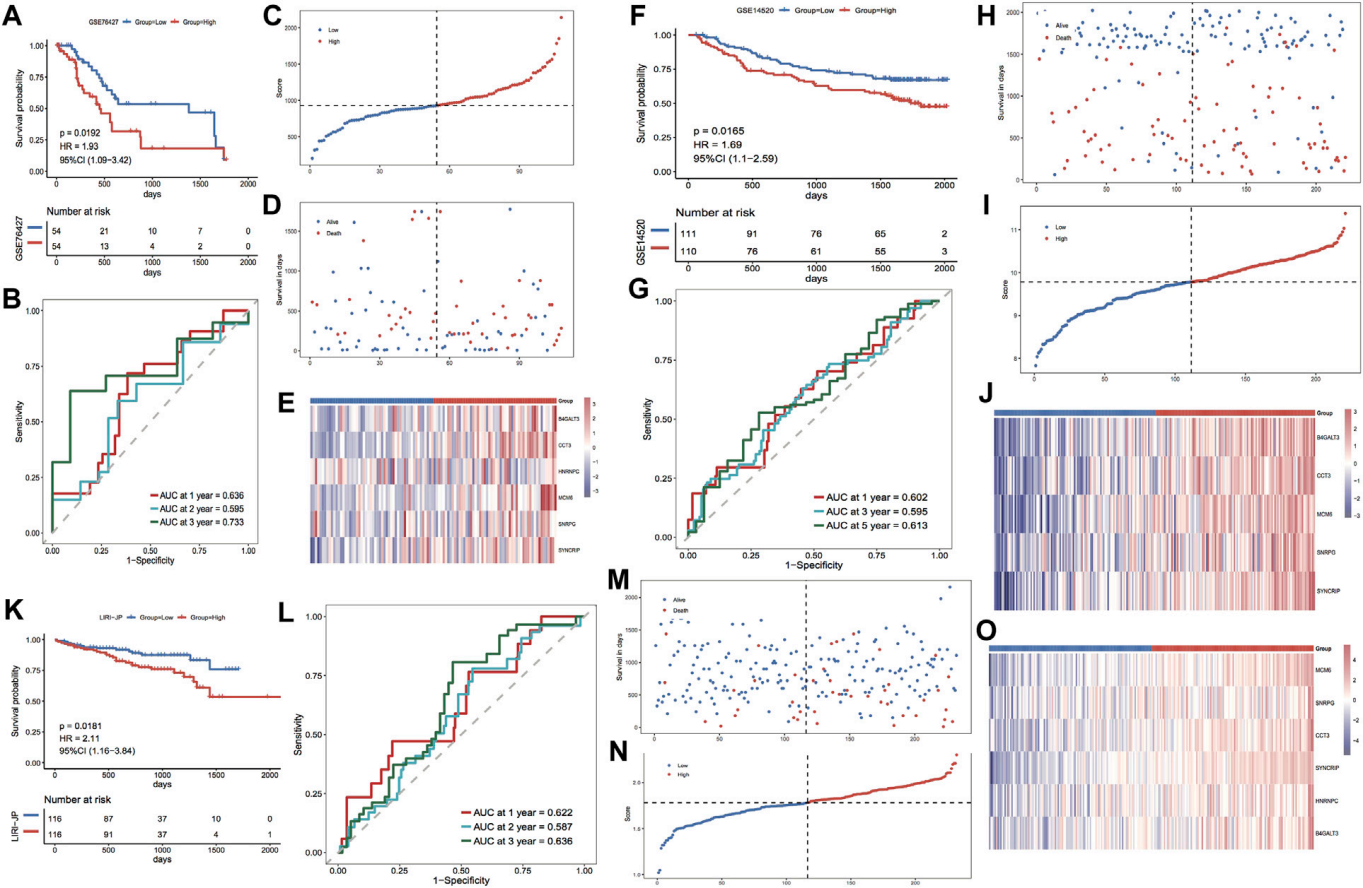
Prognostic efficacy of the validation model for the GEO dataset. **(A,B)** KM curves and ROC curves for validation set GSE76427; **(C–E)** Risk triple-plot for validation set GSE76427; **(F,G)** KM curves and ROC curves for validation set GSE14502; **(H–J)** risk triple-plot for the validation set GSE14502; **(K, L)** risk triple-plot for the validation set KM curves and ROC curves of LIRI-JP; **(M–O)** risk triple-plot diagrams of the validation set LIRI-JP.

For further validation of the model score’s strength in predicting the overall survival of individuals with HCC, three GEO external datasets were selected to proceed with the same analytical validation in this study. For the validation set GSE76427, the KM curve results showed major variations in prognosis between the two risk groups, with a worse prognosis in the high-risk group (Figure 7A). The ROC curve results showed the respective AUCs of 0.636, 0.595, and 0.733 at 1, 2, and 3 years (Figure 7B). The scatter plots of the sample risk scores, and the scatter plots of survival time and status are shown in Figures 7C,D. The thermographic of model gene expression in the validation set GSE76427 is illustrated in Figure 7E. For the validation set GSE14502, the KM curve outcomes highlighted major variations in prognosis between both risk groups, with a worse prognosis in the high-risk group (Figure 7F). The ROC curve results highlighted the respective AUCs of 0.602, 0.595 and 0.613 at 1, 2 and 3 years (Figure 7G). The scatter plots of the sample risk scores and the scatter plots of survival time, and survival status are shown in Figures 7H,I. The expression thermographic of model genes in the validation set GSE14502 is shown in Figure 7J. For the validation set LIRI-JP, the KM curve results showed significant differences in prognosis between the two risk groups, with a worse prognosis in the high-risk group (Figure 7K). The ROC curve results showed the respective AUCs of 0.622, 0.587 and 0.636 at 1, 2 and 3 years (Figure 7L). The scatter plots of the sample risk scores, and the scatter plots of survival time and survival status are shown in Figures 7M,N. The thermographic of model gene expression in the validation set LIRI-JP is shown in Figure 7O. The prognostic efficacy of the model performed well in the three GEO external validation sets, and the expression trends of the model genes were identical to those of the TCGA dataset.

### Prognostic risk model correlated with multiple characteristics of HCC

#### Clinical characteristics linked with risk scores

**FIGURE 8:**
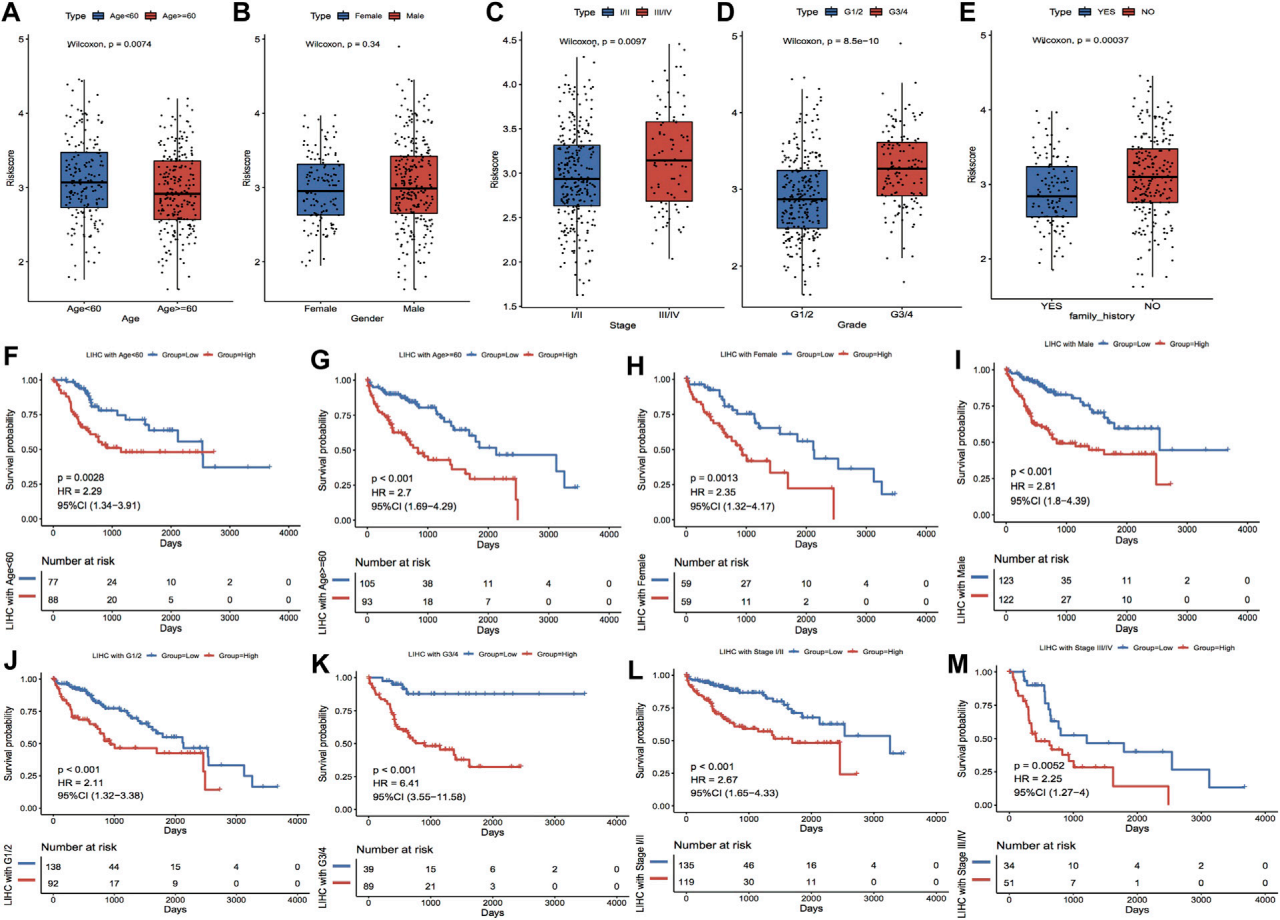
Clinical characteristics correlated with model scores. **(A–E)** show the distributions of Riskscore in the clinical characteristics grouping, corresponding to age, gender, stage, grade and family history, respectively. **(F–M)** show the subgroups of Age, Gender, Grade and Stage characteristics, respectively. In the KM curves of the dataset, red highlights the high-risk group and blue highlights the low-risk group.

Based on the clinical characteristics the TCGA_LIHC dataset, we explored the differences in Riskscore distribution among different subgroups of clinical characteristics; the outcomes highlighted that Riskscore was considerably varied in the subgroups of age, stage, grade, and family history (Figures 8A–E). In addition, based on the grouping information of age, gender, stage, and grade, the TCGA dataset was divided into two sub-datasets, and the KM curves of the sub-datasets were plotted separately according to the grouping of median Riskscore; the results showed that the KM curves of each sub-dataset were significantly different, with a worse prognosis in the high-risk group (Figures 8F–M).

#### RiskScore as an independent prognostic factor

**FIGURE 9:**
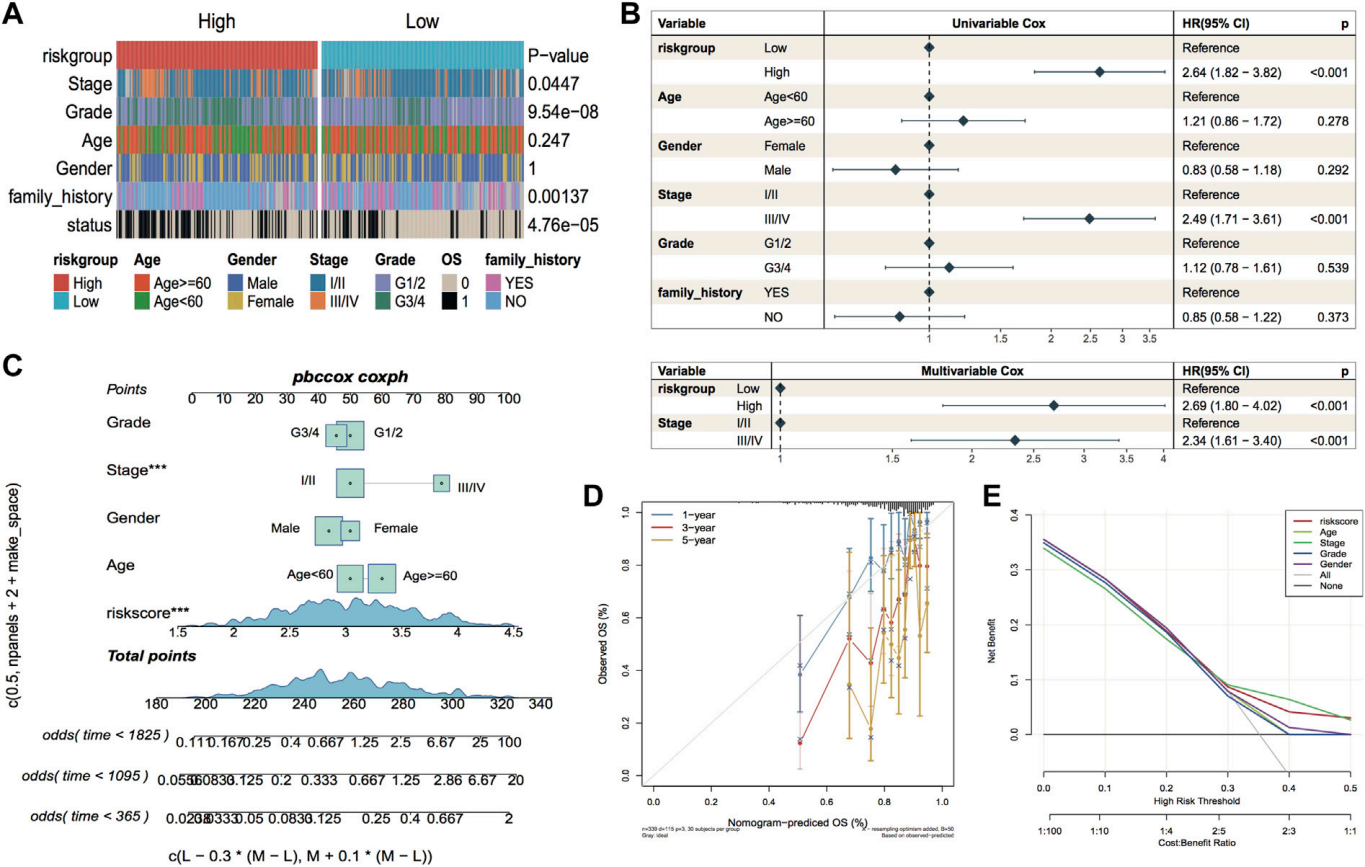
Independence of model scores in clinical characteristics. **(A)** Thermographic of clinical characteristics distribution in LIHC sample, the *p*-value is the significance of the difference between characteristics grouping compared to riskgroup grouping; **(B)** Forest plot of single-multivariate cox analysis of clinical factors in TCGA cohort; **(C)** Nomogram of the predictive model, the square plus line segment represents the size of the contribution of the clinical factor to the outcome event. Total Points represents the total score of all variables taken after the corresponding individual scores are added together, and the bottom three lines represent the probability of survival at 1, 3, and 5 years corresponding to each taken point; **(D)** Calibration curve, the horizontal coordinate is the predicted probability, the vertical coordinate is the actual probability, the closer to the middle gray line represents the more accurate predicted risk probability, below the gray line represents the underestimated risk, above The lower part of the gray line represents an underestimation of risk and the upper part represents an overestimation of risk. **(E)** Decision Curve Analysis for nomogram and other variables.

The constructed risk model showed good prognostic efficacy in the TCGA dataset and the GEO external validation set. The complex thermographic of Figure 9A shows major variations in the distribution of clinical features of stage, grade, family history, and OS of the samples in the two risk groups, indicating that these clinical factors are correlated with the model groupings. In addition, to verify whether RiskScore has the ability to act as an independent prognostic factor, a single-multivariate cox regression analysis was performed combining age, gender, clinicopathological stage, clinical grade, and family genetic history of liver disease in LIHC. In the single-multivariate cox regression, both prognostic model grouping and clinical staging were significantly different relative to the reference, demonstrating that they were independent prognostic factors (Figure 9B). In addition, a nomogram based on survival time and survival status, along with clinical indicators, showed stage and riskscore as contributing clinical factors (Figure 9C). Further calibration curve plots were drawn to assess the accuracy of the nomograms, and the outcomes revealed that the predictive accuracy of the model was high at 1 and 3 years (Figure 9D). Furthermore, DCA decision curve plots for different categorical features were used to assess the prediction accuracy of multiple clinical features; the outcomes are illustrated in Figure 9E.

#### Correlation of model grouping with the proportion of immune cell infiltration

**FIGURE 10:**
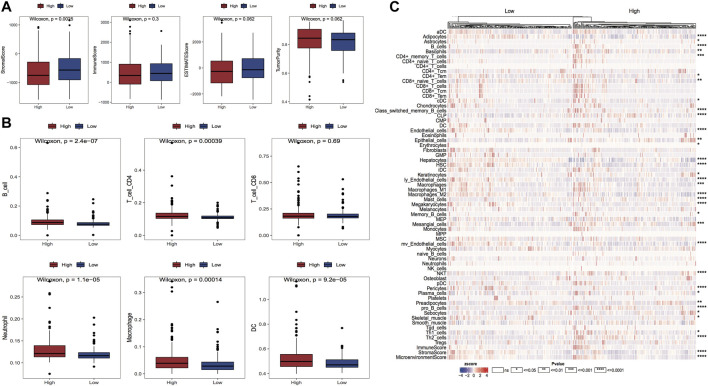
Variations in the proportion of immune infiltrating cells between model subgroups. **(A)** Box line plots of matrix score, immune score, ESTIMATE score and tumor purity for high and low-risk groups, respectively, red for high-risk group and blue for the low-risk group; **(B)** Box line plots of the proportion of immune infiltrating cells for high and low-risk groups in TIMER algorithm, red for high-risk group and blue for the low-risk group; **(C)** Thermographic of the variation in the abundance of immune infiltrating high and low-risk groups in the xCell algorithm.

In the tumor microenvironment, immune cells and matrix cells are the two main types of non-tumor components and have been shown to be valuable in the diagnostic and prognostic assessment of tumors. In this study, we calculated the immune, matrix, and ESTIMATE scores along with tumor purity; the outcomes highlighted that the matrix score was considerably reduced in the high-risk group in comparison with the low-risk group (Figure 10A). We also calculated the difference in immune cell infiltration ratio in the two risk groups using TIMER and xCell algorithms, respectively; the results are shown in Figures 10B,C. Figure 10B demonstrates the results of the TIMER algorithm for immune infiltration, in which there are five major cell types with significant differences in the percentage of immune infiltration in both risk groups and the percentage of infiltration in the high-risk group was high. The proportion of HSC cell infiltration in the high-risk group was considerably lower in comparison with that in the low-risk group.

#### Expression of model genes correlates with the proportion of immune cell infiltration

**FIGURE 11:**
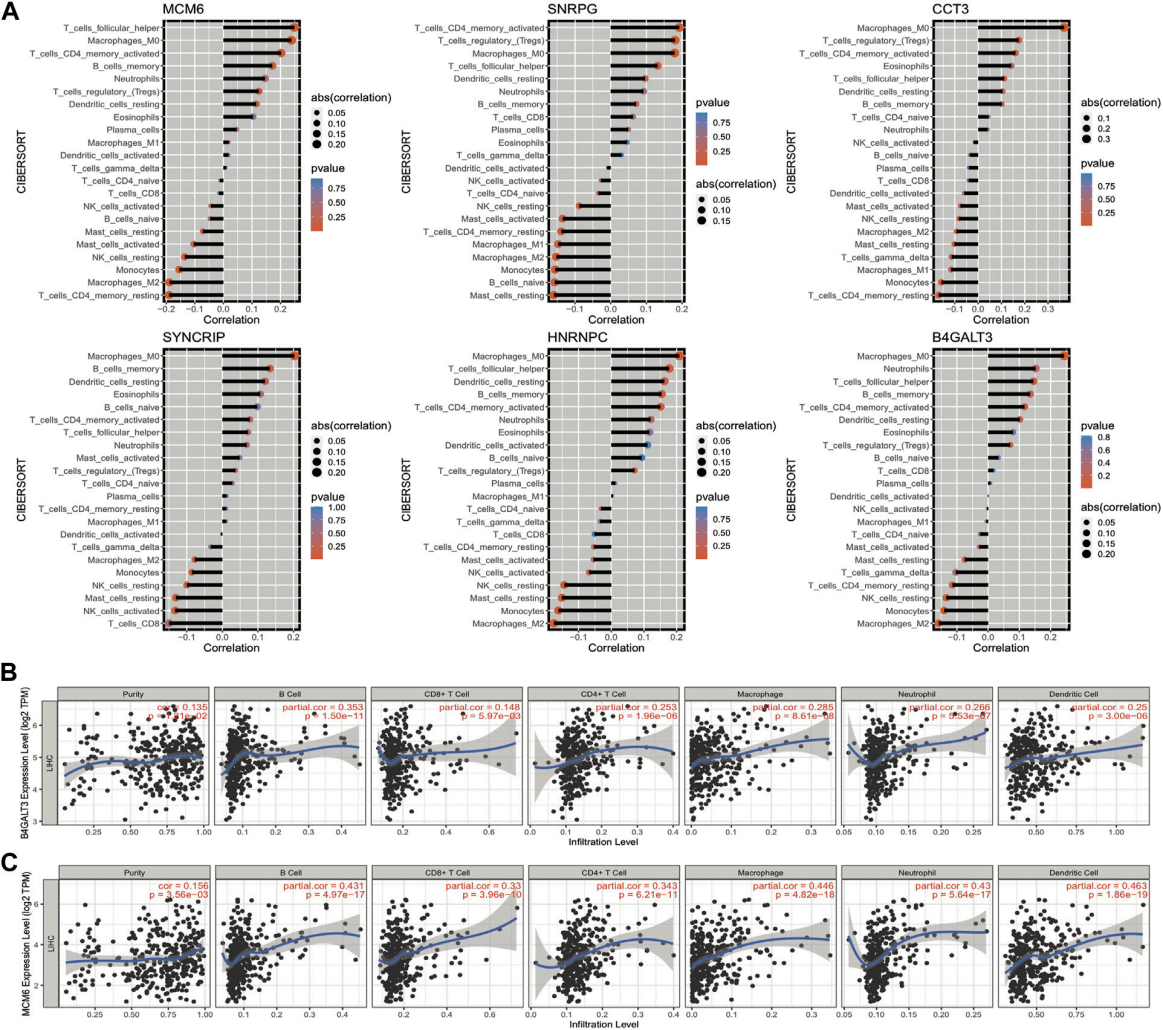
Correlation between the expression of model genes and the proportion of immune cell infiltration. **(A)** Bar graph of the correlation between model gene expression and the proportion of immune cell infiltration, the length of the bars demonstrate the size of the correlation, and the color represents the significant *p*-value of the correlation; **(B,C)** Scatter plots of correlation coefficients between the expression values of model genes B4GALT3 and MCM6 (TPM) and the proportion of immune cell infiltration obtained from TIMER online website analysis.

The grouping information of the risk model is closely linked with the expression of model genes, and we can explore how the expression of genes affects the prognosis of cancer by investigating the linkage between the expression of model genes and the immune microenvironment. The results of the immune cell infiltration ratio were calculated according to the cibersort algorithm, and the significance of gene expression in clinical immunology was represented by calculating the correlation coefficient between the expression of model genes and each immune cell infiltration ratio in LIHC samples. Six model genes and 23 immune cell infiltration ratio correlation coefficient plots are shown in Figure 11A. Moreover, we assessed the link of gene expression (TPM) with six immune cell infiltration ratios and tumor purity in TCGA data through the TIMER website, and we selected two of the model genes for presentation (Figures 11B,C); other results are shown in the Appendix.

#### Expression and clinical significance of model genes

**FIGURE 12:**
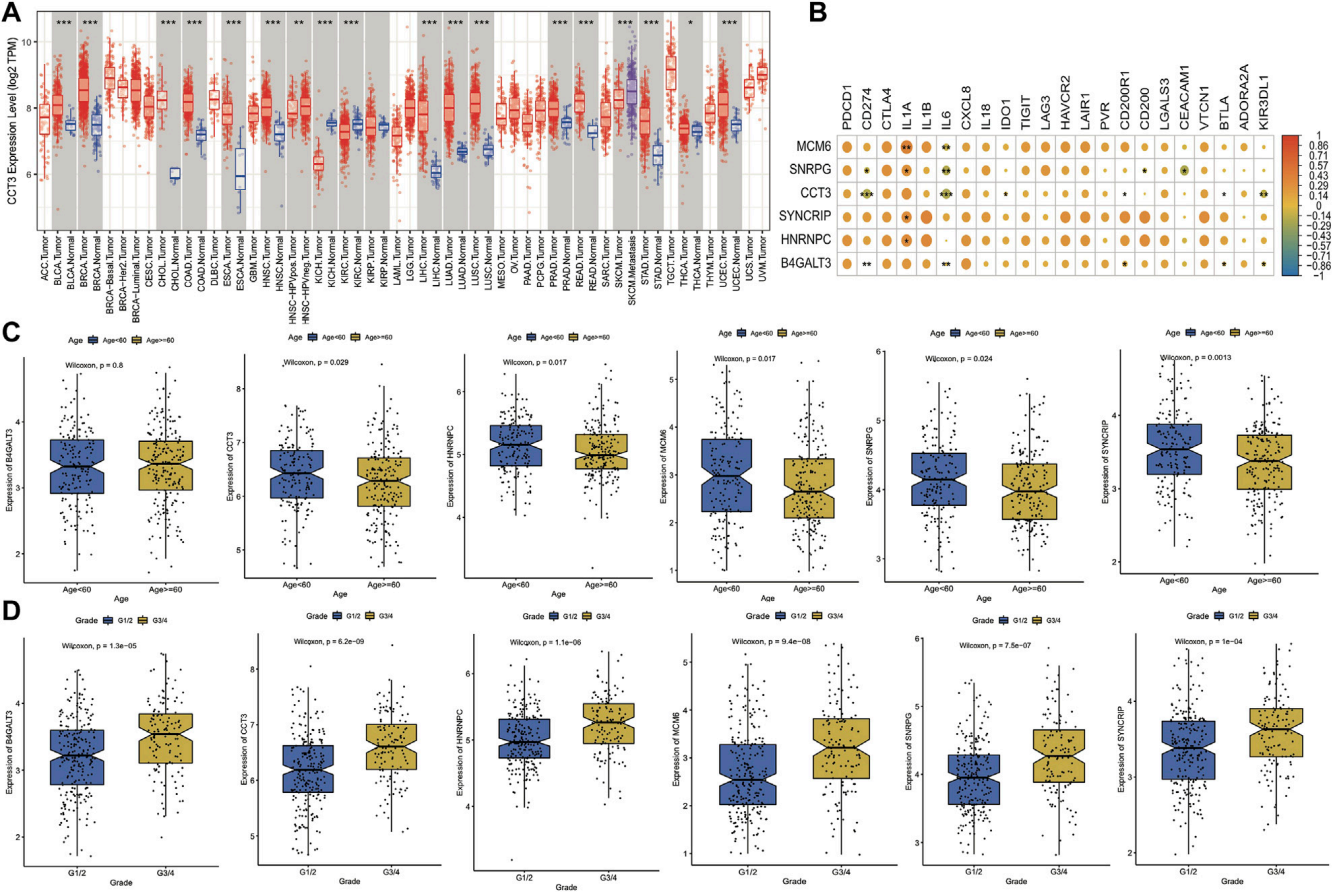
Expression and clinical significance of model genes. **(A)** Box line plot of model gene expression in pan-cancer, if Normal samples are present in the TCGA cohort for that cancer, they are also plotted simultaneously and expressed in blue; **(B)** Thermographic of the correlation coefficient between model gene and immune checkpoint expression, the color of the dot represents high correlation and * represents significance; **(C)** Box line plot of model gene expression difference in Age grouping, where blue is Age<60 and yellow is Age>=60; **(D)** Box line plot of expression differences of model genes in Grade grouping, where blue is G1/2 and yellow is G3/4.

To confirm the correlation between model genes and cancer, we analyzed and visualized the expression differences of each model gene in pan-cancer samples through the TIMER website. The box plot of CCT3 gene expression in pan-cancer is shown in Figure 12A, which is commonly up-regulated in tumor samples, and the analysis graphs of other genes are shown in the Appendix. Immune checkpoints are a series of molecules expressed in immune cells that control the degree of immune activation, and they are crucially involved in the development of human autoimmune effects. In this analysis, we selected 22 immune checkpoints expressed in this dataset for analysis and calculated the correlation coefficients between model genes and their expression; the results of the thermographic display are shown in Figure 12B. Subsequently, we observed the link between the expression of model genes and clinical properties by plotting box line plots of model gene expression in different groupings based on clinical characteristics and the expression differences of 6 model genes in the age group and grade group shown in Figures 12C,D. The outcomes highlighted major variations in the expression of 5 model genes in the age group, and all model genes in the grade group were highly expressed in the G3/4 group.

#### Genomic mutational differences

**FIGURE 13:**
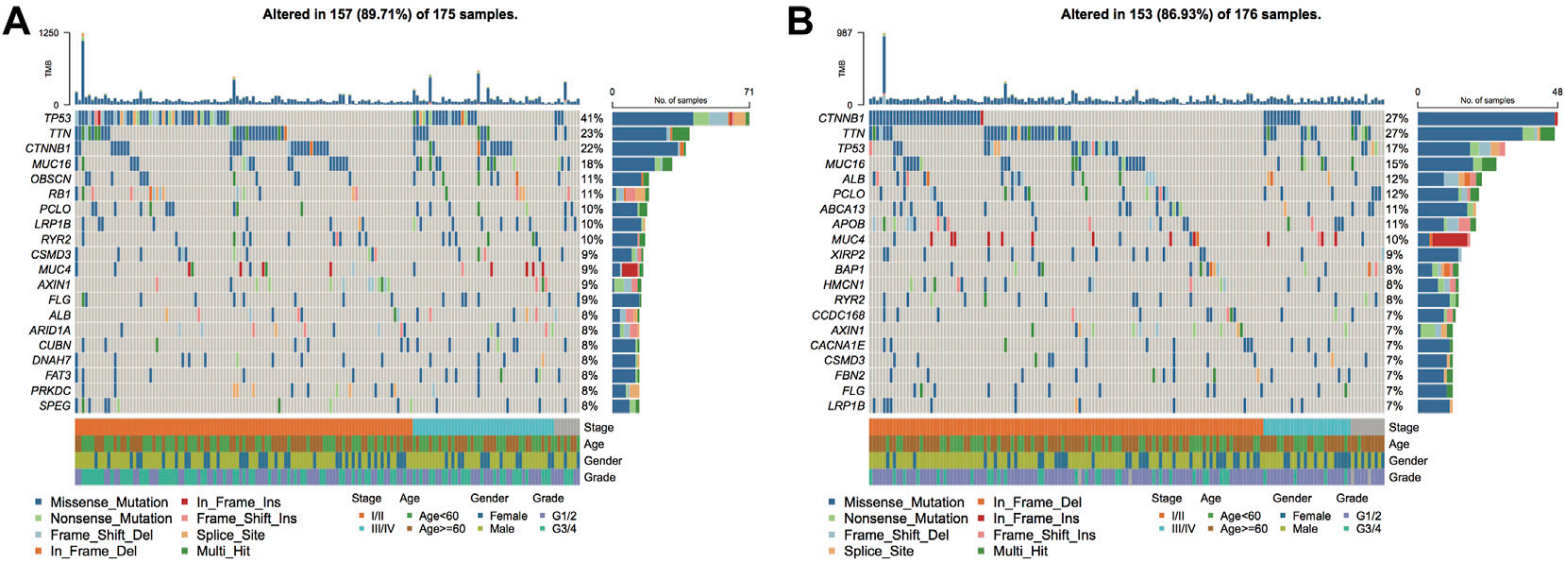
Genomic mutation differences between model subgroups. **(A)**. SNV waterfall plot of TOP20 (mutation frequency) genes in the high-risk group; **(B)**. SNV waterfall plot of TOP20 (mutation frequency) genes in the low-risk group.

Genetic mutations can stimulate cancer progression or malignant growth, and studying them at the molecular level is crucial for developing tumor-targeted drugs and novel therapies to treat cancer. To demonstrate the distribution of somatic variants between both risk groups across samples and to demonstrate the distribution of gene mutations between samples with different clinical characteristics, the 20 genes with the highest mutation frequencies in the two risk groups were selected to draw a waterfall plot, and the results highlighted that the TP53 gene had a considerably enhanced mutation frequency in the high-risk group when compared with the low-risk group (Figures 13A,B).

#### Correlation between model scores and HALLMARKER pathway enrichment

**FIGURE 14:**
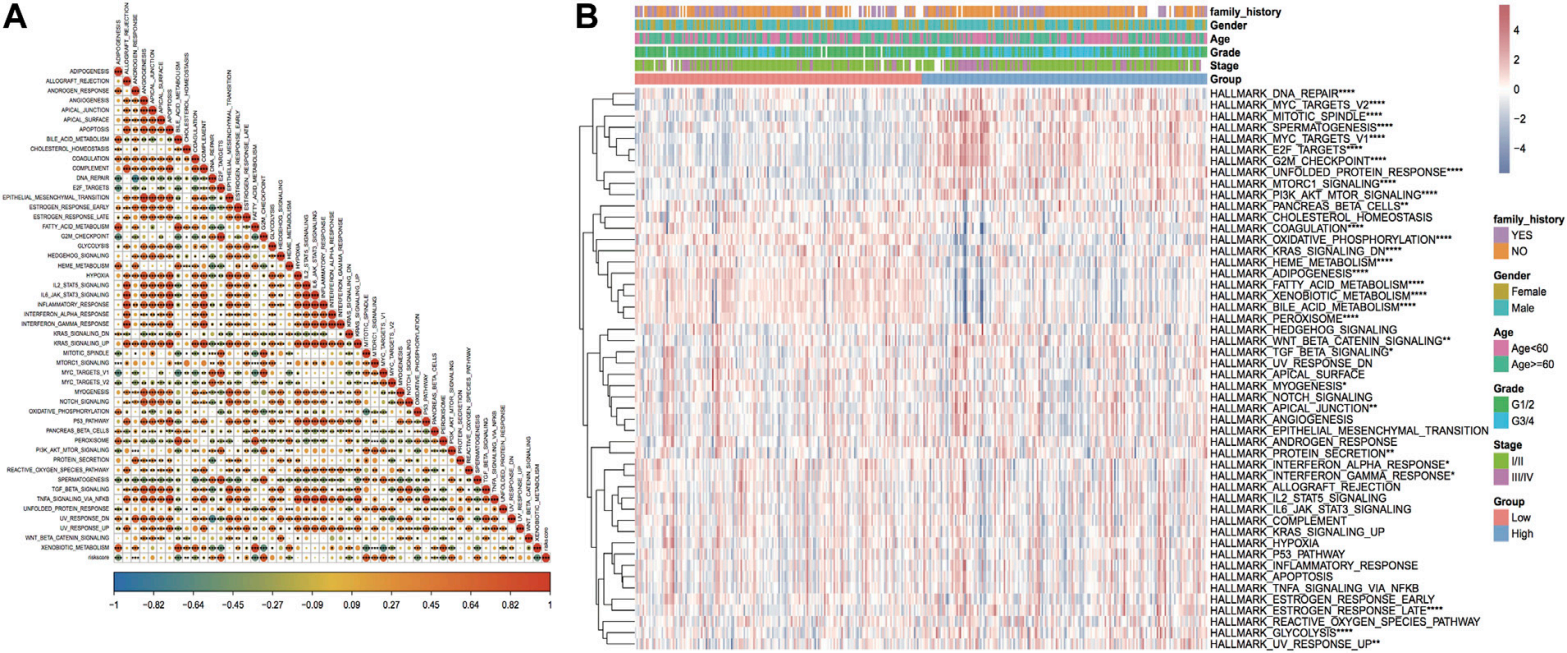
Enrichment analysis results of the HALLMARKER pathway. **(A)** Thermographic of correlation between Riskscore and HALLMARK pathway enrichment analysis, red highlights positive correlation, blue highlights negative correlation, shade represents high correlation, * sign represents significance; **(B)** thermographic of enrichment score of HALLMARK pathway, * sign represents the enrichment score of this pathway in high and low-risk groups. Enrichment score difference significance.

The results of HALLMARKER pathway enrichment scores were measured as per the expression profiles of HCC samples. Combined with the model score information, the correlation between Riskscore and enrichment score and the variation in pathway enrichment between the two risk groups are discovered, which is helpful in investigating the link between cancer characteristic pathways and prognosis. The outcomes revealed that Riskscore was considerably positively linked with five HALLMARK pathways, and six HALLMARK pathways were significantly negatively correlated (Figure 14A). Thirty pathway enrichment scores in B-plot had significant differences between model subgroups (Figure 14B).

#### Model scores to predict patients’ treatment efficacy

**FIGURE 15:**
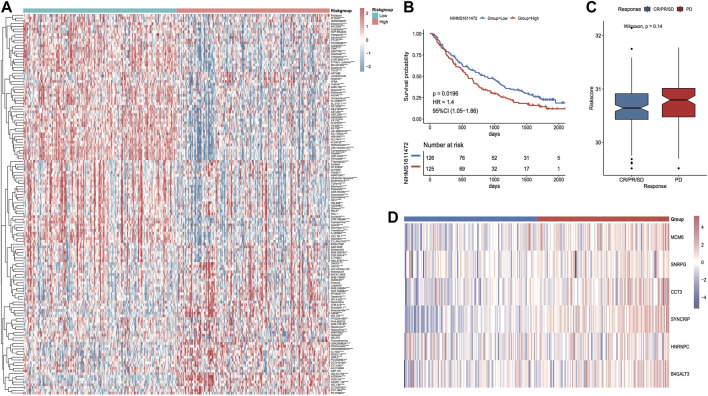
Differences in drug sensitivity between model subgroups. **(A)** Thermographic of IC50 values between high and low-risk subgroups in the TCGA LIHC cohort, red highlights high drug sensitivity and blue highlights low sensitivity; **(B)** KM curves in the immunotherapy cohort; **(C)** Box plot of risk score distribution between immunotherapy response subgroups in the immunotherapy cohort, red highlights non-response group and blue highlights response group; **(D)** Thermographic of the expression distribution of model genes in the immunotherapy cohort.

In accordance with the expression profile data of TCGA_LIHC, the sensitivity IC50 values of 138 drugs in the GDSC database were predicted. Among them, 117 drugs had major variations in IC50 values between the two risk groups (Figure 15A). In addition, to investigate whether the model genes could be used as markers of immunotherapy response, the NIHMS1611472 dataset was used to categorize the dataset into high and low-risk groups in accordance with the model risk score and plot KM curves to compare the survival differences (Figure 15B). Grouping by response information after receiving immunotherapy and comparing differences in model scores between immunotherapy response subgroups suggested that the risk scores were higher in the immunotherapy non-response group (PD) than in the response group, but the differences were not significant (Figure 15C). The thermographic of model gene expression in the immunotherapy cohort is demonstrated in Figure 15D, which indicates that the model genes are expressed increasingly in the high-risk group.

## Discussion

Surgery is currently the first choice for the treatment of primary HCC. Although surgery can remove diseased tissues, it is more invasive to operate on liver tissues adjacent to the main blood vessels, which can easily damage the important surrounding tissues and blood vessels. Moreover, most people are diagnosed in the middle or advanced stage of cancer when the surgery is most effective. Ultrasound helps significantly in the early diagnosis of individuals with HCC, and in recent years, with the promotion of minimally invasive surgery, the application of percutaneous ultrasound-guided radiofrequency ablation in the local HCC therapy has improved with time (Parizadeh et al., 2019; Selby et al., 2020). Improved microwave ablation guided by ultrasound can locate the ablation area with the assistance of ultrasound and more accurately block the arterial blood supply of tumors, thus shrinking tumors and killing tumor cells quickly (Peng et al., 2022). With less trauma, fewer complications and high reproducibility, it helps greatly in the treatment of early-stage HCC and mid- to late-stage HCC patients. In addition, recently the ultrasound medicine has broken through the limitations of traditional ultrasound imaging and has entered the “nanometer” era. For example, sonodynamic therapy (SDT) is an ultrasound-targeted activation of reactive oxygen species produced by acoustic sensitizers to kill tumors and produce immunocidal effects simultaneously (Song et al., 2018). Ultrasound-targeted microbubble destruction (UTMD), mediated by microbubbles, enables targeted delivery and tumor suppression (Tay and Xu, 2017), providing more possibilities for the treatment of HCC. Therefore, exploring the possible prognostic markers and risk models of HCC during ultrasound therapy is important for prognosis prediction and treatment of individuals with HCC.

In this study, we first performed WGCNA analysis according to the expression profile and clinical data of the TCGA LIHC cohort to identify three key modules with two major clinical features associated with HCC. The ultrasound-associated differentially expressed genes and module hub gene intersection were selected for univariate Cox analysis to identify prognostic factors significantly associated with HCC, and finally, a 6-gene signature model consisting of SYNCRIP, B4GALT3, MCM6, CCT3, SNRPG, and HNRNPC was constructed to assess HCC patient prognosis. Synaptic binding protein-binding cytoplasmic RNA interaction protein (SYNCRIP) is an RNA-binding protein that is involved in regulating biological processes such as translation regulation, mRNA stabilization, pri-miRNAs processing, variable splicing, and miRNAs compartmentalization (Mourelatos et al., 2001; Weidensdorfer et al., 2009; Geuens et al., 2016; Chen et al., 2020). Studies have shown that SYNCRIP expression can indicate a poor prognosis of HCC (Uhlen et al., 2017), and SYNCRIP can stimulate the progression of HCC by controlling the epithelial-mesenchymal transition of HCC (Riccioni et al., 2022). β-1,4-galactosyltransferase III (B4GALT3) belongs to the B4GALT family, and B4GALTs are capable of transferring galactose moieties from uridine diphosphate to oligosaccharides at the N-terminal end of acetylamino-glucose, which in turn forms acetylamino-lactose (Guo et al., 2001). Aberrant glycosylation is associated with tumor characteristics, including differentiation, adhesion, proliferation, transformation, metastasis, and tumor immunosurveillance (Brockhausen, 1999). Research has highlighted the involvement of B4GALT3 in the proliferation, invasion and metastasis of cervical cancer (Sun et al., 2016), neuroblastoma (Chang et al., 2013; Wu et al., 2020), and colon cancer (Chen et al., 2014) cells. In contrast, in HCC, highly metastatic HCC cells secrete exosomes that directly target B4GALT3, resulting in the activation of β1-integrin-NF-κB signaling in fibroblasts which in turn promotes HCC lung metastasis (Fang et al., 2018). Micro-chromosome maintenance protein 6 (MCM6) is an important factor that plays a role in initiating the replication of DNA, and it can do so after forming polymers with five other members of the MCM protein family, thus participating in the proliferation of tumor cells, and higher MCM6 expression suggests active proliferation (Zeng et al., 2021). It has been demonstrated that MCM6 has cancer-promoting effects in HCC (Liu et al., 2018a; Liu et al., 2018b). The TCPl-containing chaperone protein subunit 3 (CCT3), an important member of the chaperone protein family, is involved in protein folding and refolding (Gruber et al., 2017). It has been demonstrated that CCT3 expression is up-regulated in HCC, which in turn affects tumor progression and prognosis (Qian et al., 2016; Zhang et al., 2016; Liu et al., 2019). HNRNPC acts as an RNA binding protein and is involved in RNA splicing (Konig et al., 2010; Zarnack et al., 2013), nonspecific RNA export (McCloskey et al., 2012), RNA expression (Brunner et al., 2005), stability, and translation (Shetty, 2005). HNRNPC is up-regulated in HCC (Liang et al., 2005) and has cancer-promoting effects (Liu et al., 2022). However, there are no studies on SNRPG in HCC.

Subsequently, we validated the efficacy of the model in the TCGA training set, the overall set, and three GEO external validation sets and confirmed the risk model as an independent prognostic factor among multiple clinical indicators of HCC by single multifactor cox analysis. As per the risk score of each sample, we sorted them into high and low-risk groups, and to explore the clinical application value of the risk model, we further evaluated the percentage of immune cell infiltration, genomic mutations, pathway enrichment scores, and chemotherapeutic drug resistance differences between both groups, and the outcomes highlighted that there were major variations. For example, the frequency of TP53 gene mutations was significantly higher in the high-risk group in comparison with the low-risk group, and it has been demonstrated that in most TP53 mutant tumors, other tumor suppressor genes are similarly inactivated, and oncogenes that allow cancer progression are amplified (Donehower et al., 2019), resulting in poor prognosis, which is consistent with the worse prognosis of patients in our high-risk group. Furthermore, the poorer prognosis of patients in the high-risk group in the immunotherapy cohort suggests that immunotherapy is more effective in low-risk patients.

## Conclusion

The four key module hub genes of two major clinical features associated with HCC were identified by WGCNA analysis and intersected with ultrasound-associated differentially expressed genes to construct a six-gene signature and a risk model that can be used for prognosis prediction and immunotherapy response marker in HCC patients.

## Data Availability

The datasets presented in this study can be found in online repositories. The names of the repository/repositories and accession number(s) can be found in the article/Supplementary Material.
